# High risk of bloodstream infection of carbapenem-resistant enterobacteriaceae carriers in neutropenic children with hematological diseases

**DOI:** 10.1186/s13756-023-01269-1

**Published:** 2023-07-08

**Authors:** Li-Peng Liu, Qing-Song Lin, Wen-Yu Yang, Xiao-Juan Chen, Fang Liu, Xia Chen, Yuan-Yuan Ren, Min Ruan, Yu-Mei Chen, Li Zhang, Yao Zou, Ye Guo, Xiao-Fan Zhu

**Affiliations:** 1 Division of Pediatric Blood Diseases Center, State Key Laboratory of Experimental Hematology, National Clinical Research Center for Blood Diseases, Haihe Laboratory of Cell Ecosystem, Institute of Hematology & Blood Diseases Hospital, Chinese Academy of Medical Sciences & Peking Union Medical College, 288 Nanjing Road, Tianjin, 300020 China; 2 Tianjin Institutes of Health Science, Tianjin, 301600 China

**Keywords:** Carbapenem resistant enterobacteriaceae, Neutropenia, Children, Asymptomatic colonization, Bloodstream infection

## Abstract

**Background:**

Neutropenic children with hematological diseases were associated with higher morbidity of carbapenem-resistant enterobacteriaceae (CRE) blood-stream infection (BSI) or colonization. But it was still murky regarding clinical characteristics, antimicrobial susceptibility, and outcomes of CRE-BSI in these patients. We aimed to identify the potential risk factors for subsequent bacteremia and clinical outcome caused by CRE-BSI.

**Methods:**

Between 2008 and 2020, 2,465 consecutive neutropenic children were enrolled. The incidence and characteristics of CRE-BSI were explored in CRE-colonizers versus non-colonizers. Survival analysis was performed and risk factors for CRE-BSI and 30-day mortality were evaluated.

**Results:**

CRE-carriers were identified in 59/2465 (2.39%) neutropenic children and19/59 (32.2%) developed CRE-BSI, while 12/2406 (0.5%) of non-carriers developed CRE-BSI (*P* < 0.001). The 30-day survival probability was significantly lower in patients with CRE-BSI than in non-BSI (73.9% vs. 94.9%, *P* = 0.050). Moreover, the 30-day survival probability of patients with CRE-BSI was also poorer in CRE-carriers versus non-carriers (49.7% vs. 91.7%, *P* = 0.048). Tigecycline and amikacin exhibited satisfactory antimicrobial activity against all isolated strains. Fluoroquinolone sensitivity was lower in *E. coli* (26.3%) strains versus satisfactory susceptibility of *E. cloacae* and other CRE-strains (91.2%). CRE-BSI accompanying intestinal mucosal damage were independent risk factors for 30-day survival probability (both *P* < 0.05), while combined antibiotic therapy and longer duration of neutropenia were more prone to developed CRE-BSI (*P* < 0.05).

**Conclusion:**

CRE-colonizers were prone to subsequent BSI and CRE-BSI was regarded as an independent predictor predisposing to high mortality in neutropenic children. Moreover, individualized antimicrobial therapy should be adopted due to different features of patients with separate CRE strains.

**Supplementary Information:**

The online version contains supplementary material available at 10.1186/s13756-023-01269-1.

## Introduction

Carbapenem-resistant enterobacteriaceae (CRE) are being increasingly detected and pose a great threat to people’s health worldwide [1]. The absence of effective treatment for CRE infections turns them into life-threatening diseases [2, 3]. In China, the current result indicated that the incidence of CRE infection was about 3.0%, and the resistance rate of all antimicrobial agents had reached 11.0% in 2014 [4]. Several studies have investigated potential risk factors for CRE colonization or infection in adults, which included continual exposure to antibiotics, long-term hospital residence, indwelling medical instruments, or an immunocompromised state [5]. Despite increased attention to CRE all over the world, there is insufficient data available on the topic of CRE in children [6]. Moreover, the scientific quality of the evidence supporting CRE-bloodstream infection (BSI) management remains low. Over the past decade, CRE infections are an emerging problem in children with hematological malignancies and are associated with worse outcomes in healthcare settings [7]. The status of immunodeficiency associated with hematological diseases leads to a prolonged episode of neutropenia and these children are usually exposed to hospital admission, longer antibiotics treatment, more invasive operations and previous chemotherapy. When the effects of the factors above are on the side of disadvantages, the risk of CRE infections may soar, which in turn may further increase treatment-related mortality for children if colonized and/or infected with CRE [8]. The increasing morbidity and mortality of CRE infection in children highlight the importance of early prevention in susceptible populations and effective therapy for those infections caused by these organisms [9]. Therefore, we aim to analyze the incidence and characteristics of CRE-BSI in asymptomatic CRE-colonizers in comparison to non-colonizers in neutropenic pediatrics who suffered acute leukemia (AL) or severe aplastic anemia (SAA). What’s more, the survival analysis was performed and the potential risk factors for subsequent CRE-BSI and 30-day mortality after the onset of bacteremia were identified.

## Methods

### Study population

We included 2465 children with hematological diseases (AL and SAA) at the Institute of Hematology & Blood Diseases Hospital, Chinese Academy of Medical Sciences & Peking Union Medical College between Jan 2008 and Dec 2020. All 2465 patients were screened with perirectal swabs on the day of admission to our hospital and once weekly thereafter. The inclusion criteria of patients were: (I) age < 18 years old, (II) individuals or their caregivers who provided written informed consents. In order to reduce the influence of confounding factors in prior admission, we only included patients with newly diagnosed hematological diseases who were first admitted to the hospital. In this study, empirical antimicrobial treatment was adopted for all febrile children immediately after the collection of blood culture samples, and the use of antimicrobials was according to the guidelines of the Infectious Diseases Society of America [10, 11].

The protocol was approved by the Ethics Committee and Institutional Review Board of the Institute of Hematology & Blood Diseases Hospital, Chinese Academy of Medical Sciences & Peking Union Medical College and the study was conducted in accordance with the Declaration of Helsinki.

### Data collection and definitions

The baseline characteristics collected included age, sex, CRE strains of infection or colonization, length of neutropenia, length and type of antibiotic therapy, central venous catheter use and length of hospitalization and the concomitant gastrointestinal mucosa damage.

Enterobacterales that test resistant to at least one of the carbapenem antibiotics (ertapenem, meropenem, doripenem, or imipenem) or produce a carbapenemase (an enzyme that can break down carbapenem antibiotics) are called CRE [12]. Neutropenia was defined as the absolute neutrophil count (ANC) lower than 0.5 × 10^9^/L [13].

Recently, developed molecular assays have suggested that when compared with rectal swabs, perirectal swabs have very similar performance characteristics when used in some assays [14–17]. Allowing for the perirectal region can be considered an acceptable alternative for collecting surveillance cultures for CRE colonizing the gastrointestinal tract, we performed perirectal swabs instead of rectal swabs when the presence of neutropenia in all patients. The perirectal screening was performed for all 2465 patients on the day of admission and once weekly thereafter, and asymptomatic carrier was considered as patients with a positive perirectal swab for CRE at any one time, while who have never had a positive swab for CRE were classified as the non-colonizer. CRE blood culture was performed at the onset of a fever after hospital admission which was obtained from peripheral blood, and the onset of CRE-bacteremia was defined as the blood culture that confirmed infection of CRE [18].

The type of antibiotic therapy was categorized either as monotherapy (one antibiotic) or combined therapy (two or more antibiotics) for all previous infections but was not limited to CRE. Length of antibiotic use ≥ 14 days was defined by at least one antibiotic (cephalosporin, carbapenem or glycopeptides e.g.) used more than 14 days in the past 30 days before diagnosis of CRE-colonization or infection. Length of neutropenia was defined as the duration of ANC < 0.5 × 10^9^/L while hospitalized. According to an updated NCICTC scale (Common Terminology Criteria for Adverse Events, version 5.0 [CTCAE v5.0]), mucosal damage was measured by anatomic, symptomatic, and functional components. The symptoms of mucosal damage were associated with nausea, vomiting, bloating, diarrhea, intestinal cramping, and abdomen pain. Given that gastrointestinal (GI) bleeding can be classified as a common cause of BSI in neutropenic patients, we also included patients with GI bleeding in this study.

### Microbiological methods

CRE surveillance was performed in all 2465 patients when their hospitalization started. Allowing for the perirectal region can be considered an acceptable alternative for collecting surveillance cultures for CRE colonizing the intestinal tract, we performed perirectal swabs instead of rectal swabs when the presence of neutropenia in all patients. All swabs were transported to the central laboratory in liquid Stuart medium. In order to maximize the sensitivity of CRE screening (detection of low-level resistance and low loads of CRE), selection for CRE was conducted by inoculation onto McConkey agar plate after broth enrichment on the basis of the CDC method [19], and phenotypic antimicrobial susceptibility testing of CRE identification for isolates was confirmed using the Vitek-2 automated system (bioMérieux, Marcy-I’Étoile, France). Blood samples were cultured using a bottle and automated blood culture instrument of BD9050 (BD company, USA). Antibiotic resistance was the result interpreted according to CLSI criteria (CLSI2014) [20].

### Statistical analysis

To analyze the differences in continuous variables, a non-parametric test (Mann-Whitney U test) was used, and frequencies were analyzed using Fisher’s exact test. A logistic regression model of univariate and multivariate analysis was to evaluate risk factors for asymptomatic CRE carriers who developed BSI. Survival rates were estimated using the Kaplan-Meier method and compared using the log-rank test. In this study, we aim to investigate the effect of CRE colonization on 30-day mortality, and we consider the patients with death occurred by the end of the 30 days after the onset of CRE bacteremia or colonization as a censor. A Cox regression model was to evaluate risk factors for the 30-day survival probability of all patients. All variables with a *P* < 0.10 in univariate analysis were included in the multivariate analysis in the logistic regression model. A two-sided *P*-value of < 0.05 was deemed to be statistically significant. Statistical analysis was done using SPSS 22.0 (Chicago, IL, USA) and GraphPad Prism 6.02 software (La Jolla, CA, USA).

## Results

### Baseline characteristics

We enrolled 2465 neutropenic children in our center, and the median age was 4.5 years (range 1–16) and 1553 patients (63%) were male. CRE colonization was detected in four patients after perirectal swabs on the day of admission. Asymptomatic CRE carriers were identified in 59 of 2465 (2.39%) individuals, including 10 *Klebsiella pneumoniae* (*K. pneumoniae*), 14 *Enterobacter cloacae* (*E. cloacae*) and 35 *Escherichia coli* (*E. coli*). 19 patients of 59 asymptomatic carriers (32.2%) developed a CRE-BSI after colonization diagnosis within a median of 6–28 days (median 19 days) in the same period of hospitalization, and CRE strains in these 19 BSI patients were 3 *K. pneumoniae*, 13 *E. coli* and 3 *E. cloacae*, separately. Meanwhile, 12 patients from 2406 non-carriers (0.5%) were diagnosed with CRE-BSI, including 6 *K. pneumoniae*, 3 *E. coli* and 3 *E. cloacae* (Fig. S1). The median time from admission to the onset of bacteremia in non-colonizers, which was significantly longer than the duration of positive CRE colonization to bacteremia in CRE colonizers [47 (12–71) days vs. 19 (6–28) days, *P* < 0.001].

### Comparison between patients who developed CRE-BSI and non-BSI in asymptomatic CRE carriers

Table 1 showed the results of the comparison between asymptomatic CRE carriers with or without BSI. Of all 59 asymptomatic CRE carriers, patients who developed BSI were more likely to receive carbapenems and glycopeptides when compared with patients without BSI (both *P* = 0.030), while exposure of cephalosporin was similar between the two groups (*P* = 0.364) in the previous 30 days before diagnosis of CRE-colonization. What’s more, we found that patients with CRE-BSI had a higher proportion of combined antibiotic therapy (52.6% vs. 22.5%, *P* = 0.021), prolonged duration of neutropenia (94.7% vs. 55.0%, *P* = 0.002) and were more frequently admitted to ICU (84.2% vs. 32.5%, *P* < 0.001). However, the incidence of repeated admission and application of central venous catheter were similar between CRE-colonization patients with BSI and without BSI (*P* = 0.944 and *P* = 0.698). Notably, we found that 84.2% of CRE-BSI children suffered from severe mucositis of the digestive tract, thus patients with mucosal damage were significantly susceptible to CRE-BSI (*P* = 0.004) in asymptomatic carriers.

**Table 1:** Baseline characteristics of asymptomatic CRE carriers in children with hematologic disease

Factors	CRE-carriers who did not developed BSI (n = 40)	CRE-carriers who developed BSI (n = 19)	*P* value
Male, no. (%)	23 (57.5)	8 (42.1)	0.269
Age (years), median (range)	3 (1–13)	8 (1–13)	0.089
CRE strains, no. (%)			
*Escherichia coli*	22 (55.0)	13 (68.4)	0.561
*Klebsiella pheuminiae*	7 (17.5)	3 (15.8)	
*Enterobacter cloacae*	11 (27.5)	3 (15.8)	
Prior antibiotic exposure, no. (%)			
Cephalosporin	27 (67.5)	15 (78.9)	0.364
Carbapenem	8 (20.0)	9 (43.4)	0.030
Glycopeptides	5 (12.5)	7 (36.8)	0.030
Fluoroquinolone	3 (7.5)	2 (10.5)	0.697
Underlying diagnosis			
Acute leukemia	28 (70.0)	14 (73.7)	0.770
Severe aplastic anemia	12 (30.0)	5 (26.3)	
Mucosal damage, no. (%)	18 (45.0)	16 (84.2)	0.004
Oral mucositis	5 (12.5)	4 (21.0)	0.393
Gastroenteritis	13 (32.5)	12 (63.2)	0.026
Combined antibiotic therapy, no. (%)	9 (22.5)	10 (52.6)	0.021
Repeated admission, no. (%)	27 (67.5)	13 (68.4)	0.944
Central venous catheter, no. (%)	21 (52.5)	11 (57.9)	0.698
Admission to ICU within 60-day, no. (%)	13 (32.5)	16 (84.2)	< 0.001
Neutropenia duration ≥ 7 days, no. (%)	22 (55.0)	18 (94.7)	0.002
Status of acute leukemia			
Completed remission, no. (%)	20 (50.0)	8 (42.1)	0.355
Relapse, no. (%)	4 (14.3)	5 (35.7)	0.111

### Comparison between CRE asymptomatic carriers and non-carriers who developed BSI

On comparing the demographic data between CRE-carriers and non-carriers who developed BSI, there were obvious differences in the distribution of CRE strains (*P* = 0.005). The majority of BSI in non-carriers was caused by *K. pneumonia* (50%), and the proportion of *E. coli* and *E. cloacae* were both 25%. Unlike the non-carriers, the most common pathogen of BSI in asymptomatic carriers was *E. coli* (68.4%), while the proportion of *K. pneumonia* and *E. cloacae* were both 15.8%, which was similar to the proportion of CRE-carriers without BSI (55% for *E. coli*, 17.5% for *K. pneumonia* and 27.5% for *E. cloacae*).

No significant gender difference was found between the two groups (*P* = 0.547). Although insignificantly, the median age of patients in the non-carriers group was younger and with longer periods of neutropenia, which may indicate that non-colonized patients who suffered BSI were more likely with low immunity. Most importantly, only 6 out of 12 (50.0%) BSI patients suffered from mucositis in non-carriers when compared with 16 out of 19 (84.2%) patients in the CRE-colonization group (*P* < 0.001). (Table 2)

**Table 2:** Comparison between asymptomatic CRE carriers and non-carriers who developed BSI in neutropenic children

Factors	CRE carriers (n = 19)	Non-carriers (n = 12)	*P* value
Male, no. (%)	8 (42.1)	7 (58.3)	0.379
Age (years), median (range)	8 (1–13)	4 (1–13)	0.460
CRE strains, no. (%)			
*Escherichia coli*	13 (68.4)	3 (25.0)	0.050
*Klebsiella pheuminiae*	3 (15.8)	6 (50.0)	
*Enterobacter cloacae*	3 (15.8)	3 (25.0)	
Prior antibiotic exposure, no. (%)			
Cephalosporin	15 (78.9)	11 (91.7)	0.348
Carbapenem	9 (43.4)	6 (50.0)	0.886
Glycopeptides	7 (36.8)	4 (33.3)3	0.842
Fluoroquinolone	2 (10.5)	1 (8.3)	0.841
Underlying diagnosis			
Acute leukemia	14 (73.7)	10 (83.3)	0.531
Severe aplastic anemia	5 (26.3)	2 (16.7)	
Mucosal damage, no. (%)	16 (84.2)	6 (50.0)	0.041
Oral mucositis	4 (21.0)	3 (25.0)	0.798
Gastroenteritis	12 (63.2)	3 (25.0)	0.038
Combined antibiotic therapy, no. (%)	10 (52.6)	9 (75.0)	0.213
Repeated admission, no. (%)	13 (68.4)	10 (83.3)	0.335
Central venous catheter, no. (%)	11 (57.9)	10 (83.3)	0.140
Admission to ICU within 60-day, no. (%)	16 (84.2)	9 (75.0)	0.527
Neutropenia duration ≥ 7 days, no. (%)	18 (94.7)	12 (100.0)	0.419
Status of acute leukemia			
Completed remission, no. (%)	8 (42.1)	4 (33.3)	0.408
Relapse, no. (%)	5 (35.7)	6 (60.0)	0.239

### Antimicrobial susceptibility of separate isolates

**Fig. 1:**
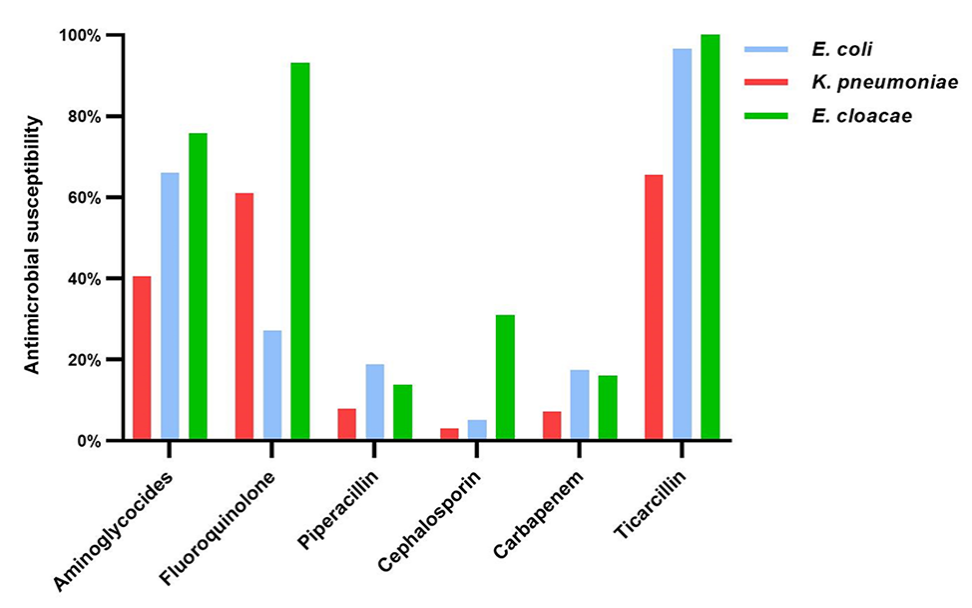
Distribution of antimicrobial susceptibility of separate carbapenem-resistant isolates

Of the 71 isolated strains from 59 CRE-colonizers and 12 non-colonizers, 38 (53.5%) were *E. coli*, 16 (22.5%) were *K. pneumoniae* and 17 (24.0%) were *E. cloacae*. Figure 1 showed the antimicrobial susceptibility of CRE isolates to six kinds of antibiotics, the antimicrobial properties to aminoglycosides, cephalosporins, tetracyclines (tigecycline), fluoroquinolones, carbapenems, and piperacillin in the *K. pneumoniae* group were 60.4%, 3.1%, 81.3%, 62.5%, 12.5%, and 6.2%, respectively. Whereas, the efficacies of these six antibiotics on strains in the *E. coli* group were 58.7%, 5.1%, 65.8%, 26.3%, 18.4% and 5.3%, separately. Nevertheless, *E. cloacae* were more sensitive to the majority of the antibiotics, especially to aminoglycosides and fluoroquinolone (77.6% and 91.2%). Even those that were extensively resistant to *E. coli* and *K. pneumoniae*, such as cephalosporin and piperacillin, 29.4% and 14.7% of *E. cloacae* were sensitive to them. (Fig. S2)

Altogether, tigecycline and amikacin exhibited satisfactory antimicrobial activity against all isolated strains. More importantly, fluoroquinolone resistance was found in the majority of *E. coli* (73.7%) strains when compared with a remarkably reduced number of *K. pneumoniae* (37.5%), together with *E. cloacae* (8.8%). Moreover, the resistance rate of piperacillin was similar to that of ertapenem (Table 3).

**Table 3:** Antimicrobial susceptibility of separate CRE isolates

Antimicrobial Agent	Susceptible strains, %		
	*Klebsiella pneumonia*	*Escherichia coli*	*Enterobacter cloacae*
Aminoglycocides	60.4%	58.7%	77.6%
Amikacin	75.0%	71.0%	94.1%
Tobramycin	56.2%	50.0%	62.1%
Gentamicin	50.0%	55.2%	76.5%
Cephalosporin	3.1%	5.1%	29.4%
Cefepime	6.2%	10.5%	52.9%
Ceftazidime	0	0	5.9%
Tigecycline	81.3%	65.8%	100%
Fluoroquinolone	62.5%	26.3%	91.2%
Ciprofloxacin	68.8%	26.3%	94.1%
Levofloxacin	56.3%	26.3%	88.2%
Carbapenem	12.5%	18.4%	25.5%
Imipenem	12.5%	23.7%	35.3%
Meropenem	18.8%	26.3%	29.4%
Ertapenem	6.2%	5.3%	11.7%
Piperacillin	6.2%	5.3%	14.7%
Piperacillin/tazobactam	6.2%	5.3%	23.5%
Piperacillin	6.2%	5.3%	5.9%

### Prognostic analysis of patients in the separated group

All children with febrile neutropenia received empirical antimicrobial therapy immediately after blood culture samples were collected. In order to minimize the emergence of antibiotic resistance, patients received a non-antibiotic therapeutic alternative (watch & wait strategy) after testing for a perirectal CRE colonization which spare them unnecessary use of antibiotics.

**Fig. 2:**
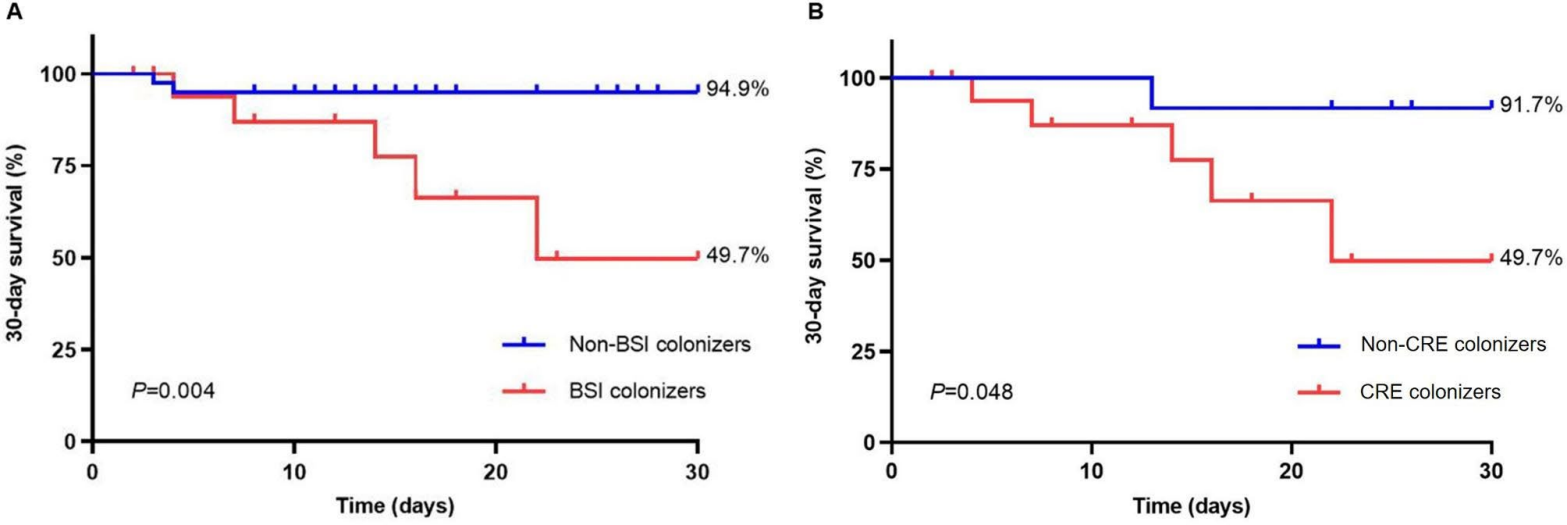
**A**. 30-day survival probability of bloodstream infection (BSI) colonizers or non-BSI colonizers. **B.** 30-day survival probability of patients with CRE colonizers or non-CRE colonizers who developed BSI

**Fig. 3:**
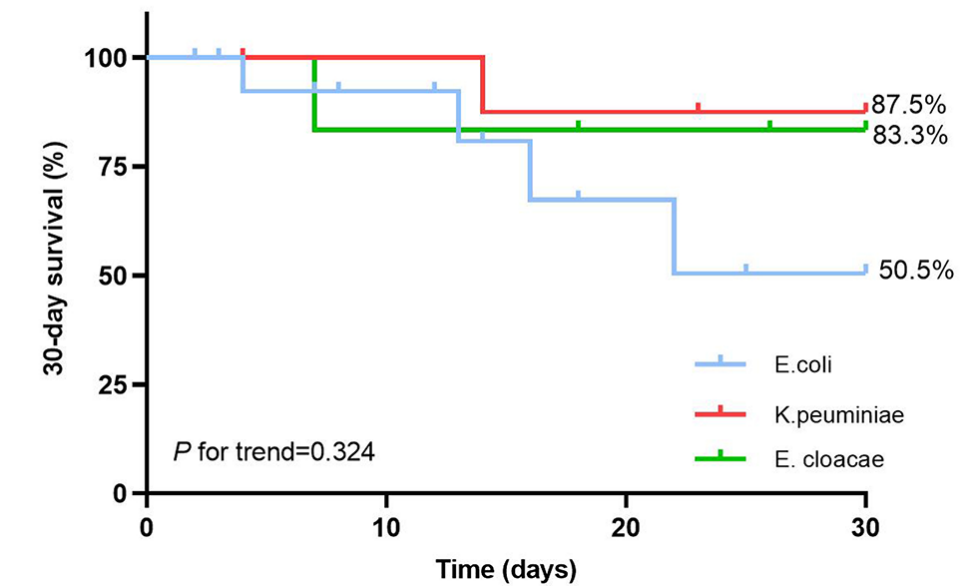
30-day survival probability of patients with separate carbapenem-resistant isolates who developed CRE-BSI

Through the survival analysis, Fig. 2A depicted that the 30-day survival probability was significantly lower in 19 children as BSI colonizers than in 40 non-BSI colonizers [49.7% (31.7–67.7%) vs. 94.9% (91.4–98.4%), *P* = 0.004]. In addition, of 31 patients with BSI, the 30-day survival probability was significantly lower in 19 CRE colonizers in comparison to 12 non-CRE colonizers [49.7% (67.7%-31.7%) vs. 91.7% (83.7-99.7%), *P* = 0.048] (Fig. 2B). As for prognosis of BSI children with separate CRE strains, the 30-day survival probability of patients infected with *E. coli* was lower than patients with *K. pneumoniae* or *E. cloacae* [50.5% (31.5–69.5%) vs. 87.5% (75.8–99.2%) vs. 83.3% (68.1–98.5), *P for trend* = 0.324] (Fig. 3).

### Risk factors for CRE-BSI and 30-day survival probability

We anatomized factors associated with 30-day survival probability after the onset of CRE-BSI (Table 4). In univariate analysis, we found a significantly higher 30-day mortality rate in children with recent admission to ICU, mucosal damage or CRE-BSI, moreover, patients with CRE colonization also took an increased risk of 30-day survival probability (all *P* < 0.05). In addition, the result of the multivariate cox regression analysis revealed that mucosal damage (*P* = 0.027), CRE colonization (*P* = 0.048) and BSI caused by CRE (*P* = 0.035) were key risk factors for 30-day survival probability.

**Table 4:** Univariate and multivariate analysis for risk factors for 30-day survival probability in all included patients

Variables	Univariate analysis		Multivariate analysis	
	HR (95% CI)	*P* value	HR (95% CI)	*P* value
CRE colonization	1.08 (1.03–1.34)	0.004	1.08 (1.01–1.15)	0.048
Bloodstream infection	10.68 (1.31–86.89)	0.027	14.32 (1.21–169.40)	0.035
Mucosal damage	9.91 (1.22–80.81)	0.032	23.87 (1.44-395.61)	0.027
Admission to ICU within 60-day	5.27 (1.06–26.15)	0.042	1.30 (0.21–8.19)	0.780

CI, confidence interval; HR, hazard ratio

When it comes to the presence of CRE-BSI, the univariate analysis demonstrated that mucosal damage, combined use of antibiotics, prolonged neutropenia duration and admission to ICU were all major risk factors for CRE-BSI (*P* < 0.05). Based on multivariate logistic regression analysis, risk factors independently associated with bacteremia in asymptomatic carriers were: mucosal damage (*P* = 0.034), duration of neutropenia ≥ 7 days (*P* = 0.007) and combined antibiotic therapy before BSI (*P* = 0.024) (Table 5).

**Table 5:** Univariate and multivariate analysis for risk factors for presence of CRE bloodstream infection in all included patients

Variables	Univariate analysis		Multivariate analysis	
	OR (95% CI)	*P* value	OR (95% CI)	*P* value
Mucosal damage	3.31 (1.22–8.96)	0.019	3.60 (1.10-11.78)	0.034
Neutropenia duration ≥ 7 days	24.55 (3.04-197.95)	0.003	19.22 (2.24-165.25)	0.007
Combined antibiotic therapy	5.45 (1.94–15.37)	0.001	3.93 (1.20-12.95)	0.024
Admission to ICU within 60-day	2.19 (0.82–5.81)	0.116	-	-

CI, confidence interval; OR, odds ratio

## Discussion

To the best of our knowledge, this is the first study represents the attempt to bring about the incidence and risk factors of asymptomatic carriers who developed CRE-BSI in neutropenic children with hematological diseases and compare the features of CRE-colonizers with non-colonizers who developed BSI. More importantly, clinical outcomes associated with CRE-BSI and potential prognostic factors on the 30-day survival probability of these patients were also investigated.

CRE is naturally resistant to most carbapenems and the optimal options for antimicrobial therapy for children suspected of having CRE-BSI are limited [21]. Previous research had explored the risk factors of CRE infected adults, nevertheless, the conditions in CRE infected children differ from those in adults. For instance, the *enterobacter* species accounted for the majority of CRE isolates in children, which was different from the reports of adult patients in which *Klebsiella* species was the most common CRE [22]. Additionally, as for the antimicrobial susceptibility of CRE isolates, in contrast to the higher rates of fluoroquinolone resistance described in the adult literature, most CRE isolates from children were susceptible to fluoroquinolone [23]. Thus, CRE strains in children may have different susceptibility to certain antibiotics in comparison to adult patients, which is due in part to the infrequent use of certain antibiotic in children which leads to less selective pressure on gastrointestinal flora and promote the development of resistance to this antibiotic. Thus, these differences showed the difficulty of translating data derived from adults into the clinical practice of children management [24].

An observational study performed by Chiotos et al. evaluated risk factors for CRE colonization or infection in pediatrics [22]. However, they did not perform further analysis in a neutropenic cohort, as neutropenia may be associated with more susceptibility to CRE infection in healthcare settings. Hence, it was relatively incomplete. An Italian retrospective study conducted by Montagnani et al. reported that CRE infection may affect immunosuppressive children with oncologic diseases who underwent chemotherapy [25]. Nevertheless, the methods of management in CRE colonized patients were not unified, thus it may indicate a problem with heterogeneity. Another study about adult data in our hospital, which have the same design and method as ours, reported that the incidence of CRE perirectal colonization was 2.54% among adults with neutropenia and CRE-BSI rate was 17.6% among 74 asymptomatic carriers [26]. Since then, few studies have analyzed the risk factors for CRE-infection among asymptomatic carriers in neutropenic pediatrics and discriminated the features of CRE-colonizers from non-colonizers who developed BSI. Given this, researches focusing on these issues may bring some illumination to the prevention of CRE bacteremia in neutropenic children with hematological disease.

In neutropenic children, an obvious difference was examined in the distribution of infecting CRE strains between carriers and non-carriers who developed CRE-BSI. The latent reason is that gut colonization of CRE potentiated the chance of pathological bacterial translocation due to increased permeability of the intestinal-vascular barrier and gram-negative bacteria, such as *E. coli*, live there in their billions. Thus, it is plausible that *E. coli* is the most common cause of BSI in asymptomatic carriers, while *K. pneumoniae* is related to the higher occurrence of BSI in non-carriers. Given the difference in the prognosis of children infected with separated CRE strains, it is crucial to highlight the challenges in specifying the type of CRE strains and combinations of antibiotics recommended for neutropenic children who suffered *E. coli* BSI, while *E. cloacae* infection may deserve de-escalation antibiotics due to better antimicrobial efficacy.

According to our study, the incidence of asymptomatic carriers was similar with adult patients in our hospital. Although with a similar incidence of colonization, subsequent bacteremia was much more common in neutropenic children, on account of weaker protective capability than adults. Moreover, higher treatment intensity in the children with acute leukemia when compared with adults may be also associated with higher prevalence of BSI in children. Thus, perirectal screening may be helpful for starting enough attention promptly in assessable susceptible patients, especially in children suffered from severe mucositis of the gastrointestinal tract. In order to improve the prognosis of CRE-colonizers, certain multimodal strategies may be suggested so as to result in a more broad-based benefit to prevent CRE perirectal colonization, like high fidelity hand hygiene, environmental cleaning and chlorhexidine bathing. Allowing for the mucosal damage was considered an independent risk factor of subsequent CRE-bacteremia and 30-day survival probability, and children with AL who received chemotherapy may lead to severe mucosa damage to the gastrointestinal tract [27]. Treatment of intestinal mucositis should be initiated as early as possible, provided that administration of antibiotics cannot be avoided, it is essential to analyze the gut microbiome changes in case of the long-term and combined usage of these agents.

There still exist limitations in this study. Firstly, although our data about risk factors were statistically significant, the study was limited by the low number of children with CRE-BSI, even if the examined cohort includes both colonized and non-colonized patients. However, this may be regarded as a positive element, suggesting a limited CRE spread in our center. The keys to success in preventing the infection of CRE are early detection through perirectal swab screening, proper hand hygiene, isolation carriers cohort, air quality control through high-efficiency particulate air filtration, as well as active surveillance measures. Secondly, allowing for the retrospective nature of the study, it is possible that there was an incomplete capture of demographic variables or clinical features if these were not documented in the electronic health record. Thus, we cannot exclude the possibility that some variables may be regarded as independent risk factors for CRE-BSI in neutropenic children.

## Conclusions

Collectively, CRE-BSI was regarded as an independent predictor predisposing to high morbidity and mortality in neutropenic children. Moreover, individualized antimicrobial therapy should be adopted due to different features of patients with separate CRE strains.

## Electronic supplementary material

Below is the link to the electronic supplementary material.

Supplementary Material 1

## Data Availability

The data that support the findings of this study are available from the corresponding author upon reasonable request.
