# Supplementary material for: High risk of bloodstream infection of carbapenem-resistant enterobacteriaceae carriers in neutropenic children with hematological diseases
Source: Antimicrob Resist Infect Control. 2023 Jul 8;12:66. doi: 10.1186/s13756-023-01269-1 (PMC10329308; doi:10.1186/s13756-023-01269-1)
Supplement: Supplementary file 1 — Supplementary Material 1 [file 13756_2023_1269_MOESM1_ESM.docx]

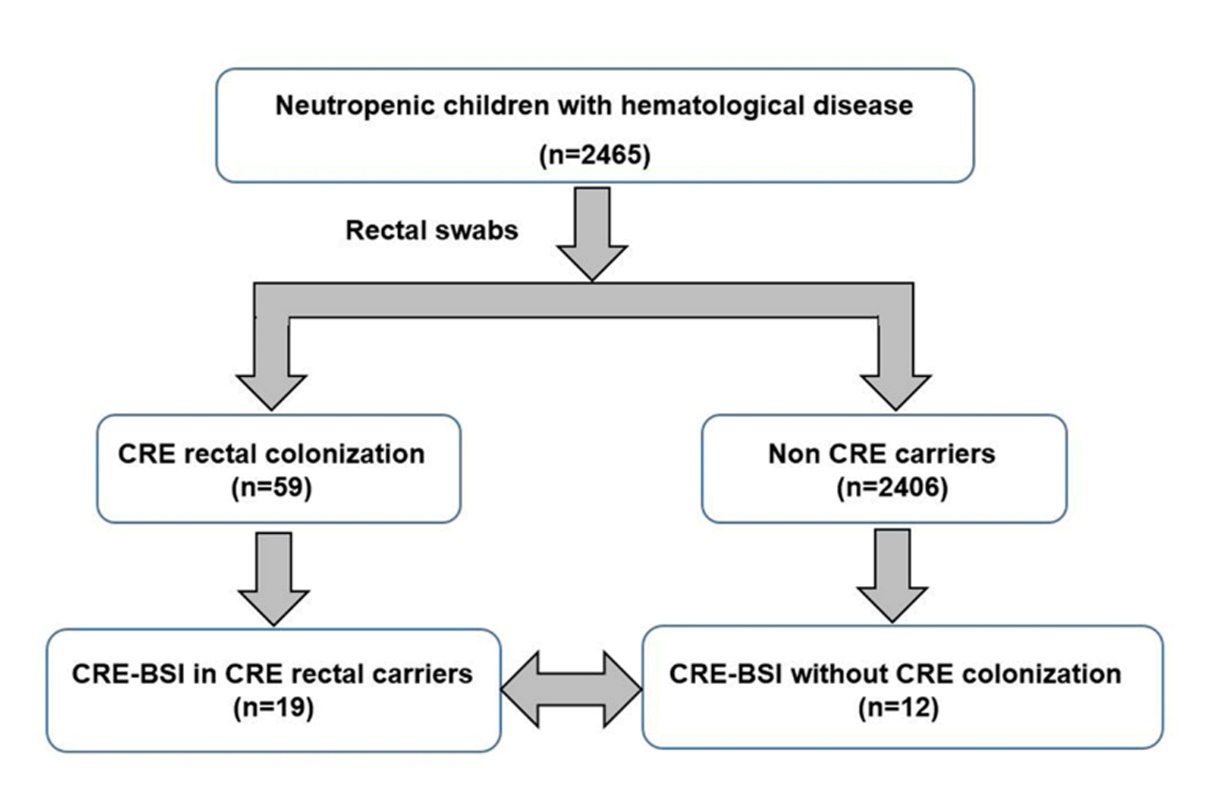


**Figure S1**. Flow diagram of the study design.


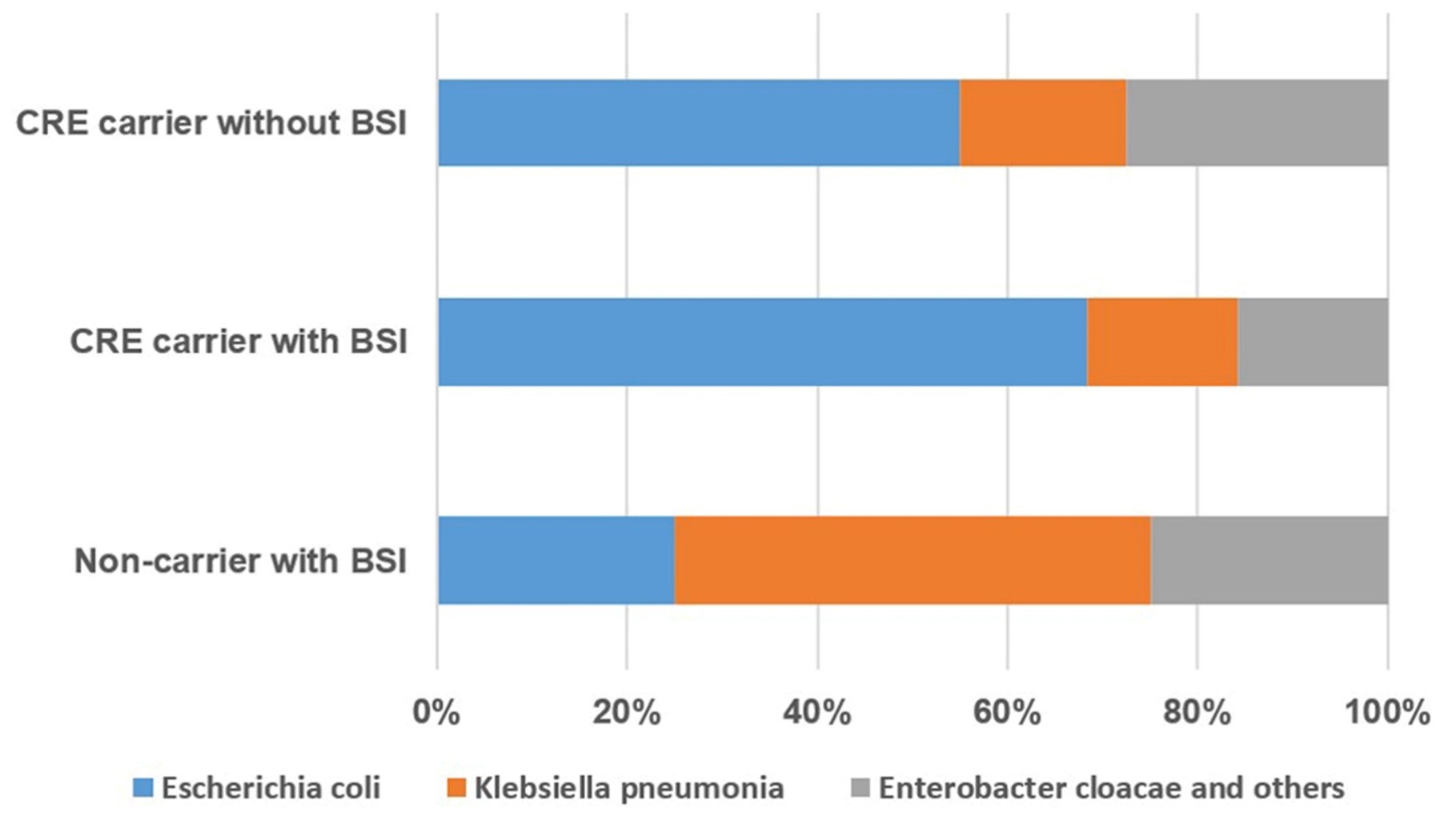


Figure S2. The distribution of the isolated CRE strains among three groups. BSI: bloodstream infection.
